# A case requiring tracheal stenting due to superior vena cava syndrome developing after craniotomy

**DOI:** 10.1186/s40981-015-0024-3

**Published:** 2015-11-05

**Authors:** Shota Sonobe, Satoki Inoue, Kazuaki Atagi, Masahiko Kawaguchi

**Affiliations:** Division of Intensive Care, Nara Medical University, 840, Shijo-cho Kashihara, Nara, 634-8522 Japan

**Keywords:** Superior vena cava syndrome, Mediastinal mass, Tracheal stent

## Abstract

We report a patient who developed sustained hypotension during craniotomy; further, owing to a mediastinal mass, critical tracheal stenosis and brain edema were observed after craniotomy, despite the absence of preoperative symptomatic superior vena cava (SVC) syndrome. A 62-year-old man underwent removal of a suspected metastatic brain tumor. The main brain tumor was speculated to be a metastatic tumor from lung cancer. A subsequent chest CT revealed a large solid tumor in the mediastinum. The maximum reduction in the cross-sectional area of the trachea was estimated to be 50 %. In addition, bilateral innominate veins were completely obstructed, and the superior vena cava was involved in the mass and was completely compressed. The patient did not show any cardiopulmonary symptoms or upper body edema. Intravenous lines were secured at the right extremity. General anesthesia was induced without any complications and was maintained with sevoflurane, remifentanil, and rocuronium. During the surgery, hemodynamic status fluctuated and was unstable. To maintain systolic blood pressure, continuous, massive infusion of noradrenaline was required. After the surgery, the patient was turned to the supine position. Massive facial edema was apparent. In addition, the bilateral upper extremities were significantly swollen. Despite the removal of the main lesion, brain edema was still observed on head CT. Chest CT revealed that the maximum reduction in the cross-sectional area of the trachea was estimated to be >90 %, which necessitated mechanical ventilation with tracheal intubation. On the day following craniotomy, tracheal stenting was performed uneventfully. The patient’s trachea was finally extubated, and his respiratory condition did not deteriorate. Although he did not develop SVC syndrome, the patient died from asphyxiation after coughing up blood at home 5 months after the procedure. It was suggested that fluid infusion from the upper extremities owing to the mediastinal tumor caused critical SVC syndrome.

## Background

In case of impaired blood flow through the superior vena cava (SVC) to the right atrium due to external compression or intrinsic obstruction of the SVC, it is recommended that intravenous access should be secured in the lower rather than in the upper extremities for anesthesia management [1, 2]. However, to the best of our knowledge, there is no report available that describes such patients developing SVC syndrome perioperatively. We report a patient who developed sustained hypotension during craniotomy; further, owing to a mediastinal mass, critical tracheal stenosis and brain edema were observed after craniotomy, despite the absence of preoperative symptomatic SVC syndrome.

## Case presentation

**Fig. 1:**
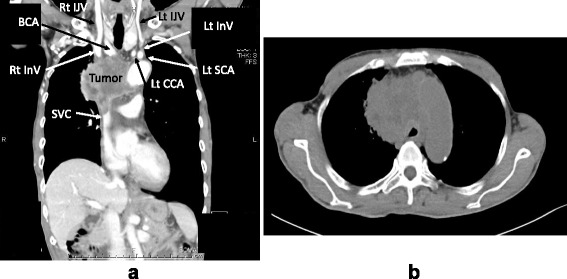
Contrast-enhanced CT findings. **a** A coronal slice showing a large mass (maximum diameter, 72.25 mm) in the mediastinum, which completely interrupts bilateral innominate veins. **b** An axial slice showing that the mass compresses the trachea with significant reduction of the tracheal cross sectional area by approximately 50 %. InV; innominate vein, IJV; internal jugular vein, BCA; brachiocephalic artery, CCA; common carotid artery, SCA; subclavian artery, SVC; superior vena cava

A 62-year-old man was admitted to our hospital for the removal of a suspected metastatic brain tumor. He had suffered from gait disturbance and visual field defects for 3 months. He has recently developed left hemiplegia due to multiple brain lesions. He had no specific past history aside from being a heavy smoker. The main brain tumor was located in the right occipital lobe, and the imaging study suggested that it was a metastatic tumor from lung cancer. A subsequent chest CT revealed a large solid tumor (maximum diameter, 72.25 mm) in the mediastinum (Fig. 1a). As for the main bronchus, the maximum reduction in the cross-sectional area was estimated to be 50 % in the side just rostral to the bifurcation (Fig. 1b), and the length of the stenosis was approximately 35 mm. In addition, the bilateral innominate veins were completely obstructed before merging into SVC, and SVC was involved in the mass and was completely compressed. Despite the imaging findings, he did not show any cardiopulmonary symptoms or upper body edema. Initially, we intended to consult a pulmonologist for further examination before craniotomy; however, the removal of the tumor was prioritized because of progressive neurological symptoms.

Peripheral 20-G and 22-G intravenous lines were secured at the right forearm and the right back of the hand. General anesthesia was induced with 100 mg of propofol plus 100 μg of fentanyl, and neuromuscular blockade was achieved with 50 mg of rocuronium. Manual ventilation with a mask was easily performed. Tracheal intubation with a 7.5 mm silicone reinforced tracheal tube was performed uneventfully. Before tube fixation, we confirmed that the distal tube end was positioned to avoid injury to the stenotic part of the trachea using a fiberoptic bronchoscope. General anesthesia was maintained with 1–1.5 % of sevoflurane, 0.05–0.1 μg/kg/min of remifentanil, and 10–15 mg/h of rocuronium. Transient hypotension was observed during induction of the anesthesia; however, it was treated using 8 mg of ephedrine. Craniotomy and removal of the brain tumor was performed in the left park-bench position. During the surgery, hemodynamic status fluctuated and was unstable, which became particularly apparent after an increase in urine output by mannitol administration. To maintain systolic blood pressure at 80–90 mmHg through the procedures, continuous, massive infusion of noradrenaline (0.05–0.07 μg/kg/min) was required. The operation time for the procedures was prolonged to 4 h and 25 min probably because of poor brain relaxation. Fluid balance including crystalloids and colloids infusion (5600 ml), blood products transfusion (560 ml), urine output (2650 ml), and blood loss (625 ml) was 2885 ml.

**Fig. 2:**
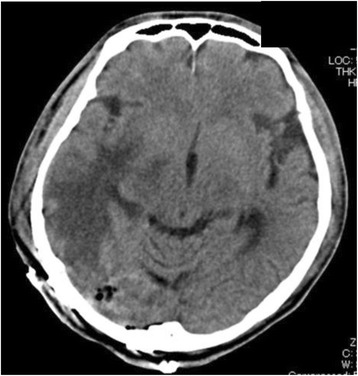
Head CT after removal of the brain tumor. Significant brain edema was confirmed in the head CT after the surgery although the right temporooccipital tumor had been removed

**Fig. 3:**
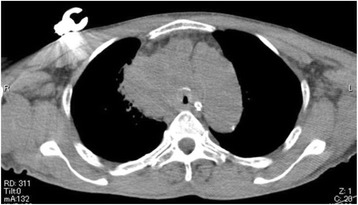
Chest CT after craniotomy. Chest CT showed that the maximum reduction in the tracheal cross-sectional area was estimated to be >90 %

After the surgery, the patient was turned to the supine position. Massive facial edema was apparent, and the bilateral upper extremities were significantly swollen. Immediately, a bronchoscopic examination was performed to evaluate the patency of the trachea, which revealed a worsened stenotic lesion. Contrary to our expectation, the respiratory condition of the patient was not impaired. Although tracheal stenting may have been a therapeutic option for this condition, we hesitated to select this option because of lack of detailed information regarding the mediastinal tumor. After the administration of 200 mg of sugamadex, the patient’s spontaneous breathing was sufficient; however, he did not regain consciousness. The tracheal tube was then removed. Critical strider accompanied with tachycardia and hypertension was observed. It did not seem likely that the patient’s respiratory condition would rapidly improve. Consequently, reintubation was performed to maintain patency of the trachea, which was confirmed by a subsequent bronchoscopic examination. The tracheal wall was supported by the tracheal tube although the tracheal tube did not pass the stenotic lesion. A head and chest CT was performed before the patient was transferred to the intensive care unit (ICU). Head CT revealed remaining brain edema with mild midline shift despite removal of the main lesion (Fig. 2). Chest CT showed that the maximum reduction in the cross-sectional area of the trachea was estimated to be >90 % (Fig. 3). Compared with the size of the tumor before the surgery, the tumor at the almost same CT slice level appeared to increase in size (Fig. 1-b and Fig. 3).

**Fig. 4:**
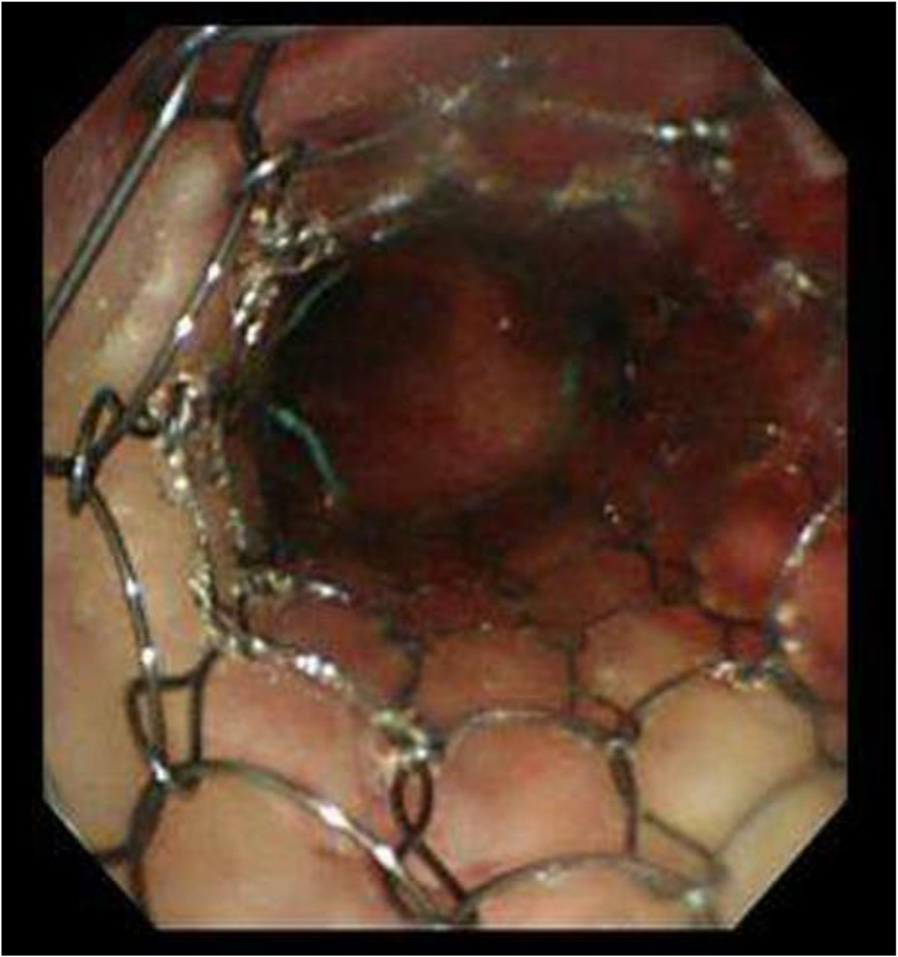
Fiberoptic bronchoscopy during tracheal stenting. Tracheal stenting was performed uneventfully

After admission to ICU, the patient remained sedated under mechanical ventilation. He was placed in the Fowler’s position to facilitate blood flow from the upper body. Simultaneously, diuretics (mannitol and furosemide) were used for edema treatment. Owing to the intensive care, the stenotic lesion improved dramatically. On the day following craniotomy, considering the progressive nature of the mediastinal tumor, tracheal stenting using a covered metal stent 60 mm long (Ultraflex™, Boston Scientific Japan, Tokyo) was performed (Fig. 4). Following the successful tracheal stenting, the patient regained consciousness immediately after cessation of sedatives. His trachea was extubated, and his respiratory condition did not deteriorate. Finally, he was discharged from ICU and underwent chemotherapy and radiotherapy. He was discharged from the hospital 3 months after the surgery. Although the patient did not develop SVC syndrome, he died from asphyxiation after coughing up blood at home 2 months after being discharged.

## Discussion

SVC syndrome results from impaired blood flow through SVC to the right atrium. Because the bilateral innominate veins were completely obstructed and SVC was involved in the mass and completely compressed in this case, SVC syndrome may have occurred at any moment preoperatively in this patient. However, the severity of the syndrome depends on the rapidity of the onset of obstruction and its location [3]. When the obstruction is slowly progressive, collateral pathways develop and symptoms are usually mild or absent, as in this case [4]. Therefore, we need to recognize that we underestimated his condition because he did not show clinical symptoms and signs of SVC syndrome.

Presuming that the respiratory and cardiovascular effects of his mediastinal tumor were not critical in the preoperative state, the reason why he developed SVC syndrome was unclear. It was an incident that would ultimately occur. Obviously, the primary cause of the rapid development of SVC syndrome was fluid infusion from an upper extremity. The fluid infusion from the upper extremity easily exceeded the drainage capacity of the collateral pathways, which increased venous pressure and promoted upper body edema. Simultaneously, the reduction of preload by mannitol-induced urination and afterload by anesthesia was not corrected by regular fluid infusion protocol using upper extremity fluid lines, which required further fluid infusion and administration of noradrenalin. This vicious circle exacerbated tracheal compression by the mediastinal tumor and brain edema. Therefore, intravenous lines in the upper extremities are contraindicated in cases with presence of SVC obstruction [1, 2] and such a patient can actually develop SVC syndrome with upper extremity fluid lines. We were overly optimistic about this case.

## Conclusions

In conclusion, we presented a case of a mediastinal tumor developing critical SVC syndrome due to fluid infusion from the upper extremities.

## Consent

Written informed consent was obtained from the patient for publication of this case report and any accompanying images. Institutional review board approval was also obtained (No.643). A copy of the written consent is available for review by the Editor-in-Chief of this journal.

## Abbreviations

SVC: Superior vena cavaIntensive care unit
ICU: Intensive care unit
CT: Computed tomography
